# The effects of straw mulching combined with nitrogen applications on the root distributions and nitrogen utilization efficiency of summer maize

**DOI:** 10.1038/s41598-020-78112-9

**Published:** 2020-12-03

**Authors:** Wan-feng Zhang, Shu-qing Yang, Ya-hong Jin, Peng Liu, Shuai Lou

**Affiliations:** Water Conservancy and Civil Engineering College, Inner Mongolia Agricultural University, Huhhot, 010018 China

**Keywords:** Plant physiology, Agroecology

## Abstract

To provide an appropriate tillage fertilization model for improving N utilization efficiency and increasing production, the field experiments were conducted to study the effects on root distributions and N utilization efficiency of summer maize involving different straw mulching modes combined with N fertilization. No (N0), low (N1), medium (N2), and high (N3) levels of N fertilization were incorporated into soil combined with the surface coverage straw (Treatment B) and the deeply buried straw (Treatment S). The traditional cultivation was used as control treatment. The results shown that treatments S had significantly promoted deep root growth, and the root length density (RLD) increased with increases in N application rate. SN2 and SN3 treatments’ average RLD were significantly increased by 67.5% and 68.1% in the greater than 40 cm soil layers. While the Treatment B had significantly increased the RLD in 0 –30 cm soil layers only. With increases in N application rate, the effect on summer maize yields increase under Treatment B were not significantly, and only BN3 increased by 0.4%, while under Treatments S were found to first increase, and then decrease. The apparent recovery efficiency of applied N, N uptake and summer maize yield of SN2 had increased by 66.8%, 20.4%, and 9.3%. Therefore the rational tillage fertilization model was deeply buried straw combined with medium N fertilizer in Hetao Irrigation District.

## Introduction

In recent years, the crop straw amounts in Hetao Irrigated District have increased year by year, and low utilization rates have been observed. And the environmental pollution issues caused by the burning of crop straw have become increasingly serious. Therefore, as an effective farming measure^1^, straw returned would not only alleviate environmental problems caused by incineration, but also improve farmland productivity and soil quality. These positive actions could play important roles in the sustainable development of agricultural resources. The results of previous related studies have shown that the returned straw could reduce evaporation, also activate valuable soil nutrients^2^. When combined with the appropriate fresh or brackish water, it had improved water productivity levels and reduced the salt content in ploughed layers^3^. The returned straw was found to regulate soil temperatures, and the larger the returned straw mulching amount was, the more obvious the cooling results would be^4^, which had obvious promoting effects on summer maize roots^5^. Roots are plants’ most active organs, and are used to absorb both water and nutrients^6^. The returned straw not only improves the roots distributions but enhances the roots ability to absorb deeply buried water and nutrients, which are particularly important links in the formation of crop yields^7,8^. Previous studies have shown that more than 85% of crop roots are distributed in the 0 to 40 cm soil layers, and at below 20 cm root layers contribute to 48% of the crop yields^9,10^. The root distributions are greatly affected by the soil ecological environmental, such as soil water and fertilizer^11^. As an important nutrient for crop growth, N affects both crop yields and quality. The main source of N for cultivated plants is through fertilizer application. In agricultural production, large amounts of N have been blindly applied for improving crop yields. However, such practices have not only failed to improve crop yields, but have easily led to excessive N content levels in soil^12,13^. Subsequently, agricultural ecological environments have faced pollution risks, and the N utilization efficiency has been reduced^14^. Wang et al.^15^ indicated that one-time applications of N during the seedling stage could better coordinate the supply of N during growth stage, and promote the absorption and utilization of N. Meanwhile, when fertilization was carried out in stages during the growth periods, and appropriate N applications were administered during the grouting periods, significantly increases both yields and N utilization rates could be achieved^16^. Previous studies^17,18^ found that of excessive N fertilizer applications were not conducive to the transfer of N and phosphorus to seeds. It was observed that the yield had not significantly increased, although serious pollution risks to the farmland environments had been caused. The results indicated that reasonable N applications could promote inter-cropping crop yield increases and effectively improves the water use efficiency^19^. Bi et al.^2^ found that reduced inter-plant evaporation had occurred regardless of whether irrigated fresh or brackish water was used under covered surface straw mulching. Wang et al.^20^ and Li et al.^21^ both determined that straw mulching practices could significantly increase soil organic carbon content levels. It found that straw mulching significantly reduced soil temperatures and straw mulching combined with suitable N fertilizer applications could improve the soil nutrients, N utilization efficiency, and economic benefits of the crops^22,23^.

However, there have been few studies conducted regarding the effects of high efficiency N fertilizer on the rhizosphere regulation of crops under straw mulching conditions in arid areas at present. The previous studies have mainly focused on the effects of only mulching or only N fertilizer applications on crop yields, and water use efficiency and N utilization. Meanwhile, few studies have considered the effects of rhizosphere regulation on the efficiency of N fertilizer under the combined actions of straw mulching and N fertilizer applications. Therefore, this study was a breakthrough point, in that various modes of straw mulching combined with different amounts of nitrogen fertilizer applications were explored. The study’s field experiments were carried out in Hetao Irrigation District of Inner Mongolia, China. The experiments examined the effects of straw mulching combined with N fertilizer applications on root distributions, the N utilization efficiency, and summer maize yields. And the interaction mechanisms among the straw mulching modes, N fertilizer applications, and rhizosphere regulation were investigated. The results obtained in this study potentially provide some useful references for future improvements in fertilizer utilization efficiency and increased crop yields under the straw mulching combined with reasonable N fertilizer applications, which will enrich the current theories regarding returning straw to soil in similar agricultural areas.

## Materials and methods

### Experiment site description

**Figure 1:**
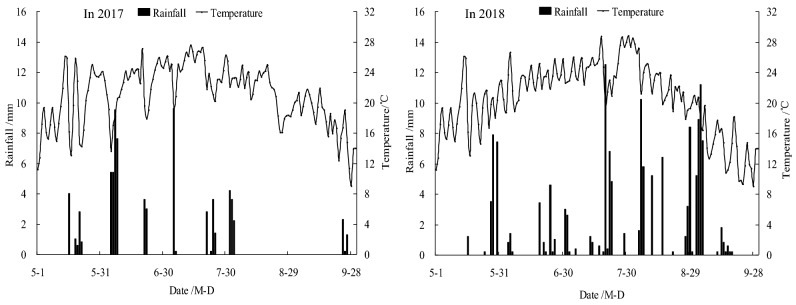
Daily rainfall and temperature changes during the growing period of the summer maize in 2017 and 2018.

The experiment plot was located in Jiuzhuang demonstration area, Hetao Irrigation District, Inner Mongolia, China (40° 42′ N, 107° 24′ E, 1040 m altitude). The plot had the characteristics of a semi-arid continental climate, with an average annual evaporation rate of 2332 mm. The surface accumulations of salt were observed to be serious during spring and winter seasons. The test soil was determined to be silty loam. The analysis results of the soil samples obtained from the same experimental area in April of 2017 revealed that the top 100 cm of soil were characterized as follows: Organic matter was 15.33 g·kg^−1^; total nitrogen was 0.87 g·kg^−1^; available phosphorus was 14.66 mg·kg^−1^; and available potassium was 180.8 mg·kg^−1^. The average bulk density of the soil was 1.51 g·cm^-3^. This study’s experiments were carried during the period ranging from April of 2017 to October of 2018. The total rainfall during the growth period of summer maize in 2017 and 2018 were 75.3 mm, and 126.9 mm, respectively. In 2017, rainfall during the growth period mainly concentrated in early June and early August, and the rainfall mainly concentrated in early July and late August in 2018. The observed daily rainfall and temperature changes in experimental area during the growth stage of the summer maize are detailed in Fig. 1.

### Experimental design

In this study, 2 factors were investigated: straw covering depth and N application amounts (pure N content, converted into urea amounts during the application process). The factors of the N applications were set as 4 specific treatments, which included no N application (treatment N0), low N application rate of 135 kg·hm^−2^ (treatment N1), medium N application rate of 180 kg·hm^−2^ (treatment N2), and high N application rate of 225 kg·hm^−2^ (treatment N3, local nitrogen application rate as 225 kg·hm^−2^). The factors of straw mulching depth were 2 set specific treatments, namely surface cover straw mulching (Treatment B, in which the farmland was turned over to 35 cm, and in second year leveling the farmland, mechanical shallow rake, roller grinding, then use machinery for film-coated planting, finally surfaces were covered with 5 cm thick straw in rows), and deeply buried straw mulching (Treatment S, in which the farmland was turned over to 35 cm, and 5 cm thick straw layers were manually applied following the autumn harvest. Then, the farmland was raked shallowly and compacted during the second year). The traditional cultivation was used as control treatment (Treatment CK, after the autumn harvest in the previous year, the mechanical tilling was about 35 cm, and a large amount of water was used for autumn-irrigation. In second year, leveling the farmland at early May, mechanical shallow rake, roller grinding, then use machinery for film-coated planting. No other field operations were performed, no straw was used, and the N application amount was 225 kg·hm^−2^). There were nine treatments (Table 1), each of which was repeated 3 times and randomly arranged. Each plot contained a 3 m protective belt and measured 72 m^2^. The surrounding plots were separated by polyethylene plastic film at buried depth of 1.2 m, with 30 cm of the film left at the top to prevent the water and fertilizer from channeling. The previous crop was summer maize in the experimental field. The crushed straw came from the ground straw of local summer maize after harvest, and the weight of straw mulching was 1.5 kg·m^−2^ and the thickness was 5 cm. The test plots adopted the management modes of the local farmers.

**Table 1:** Experiment treatments.

Treatments	Straw mulching mode	N application amount/(kg·hm^−2^)	P and K application amount/(kg·hm^−2^)	Irrigation quota
CK	Traditional cultivation	225(N3)	The P-fertilizer was DAP, and the amount was 150 kg·hm^−2^ (calculated as P_2_O_5_). K-fertilizer was KCl, and the amount was 45 kg·hm^−2^ (calculated as K_2_O). P-fertilizer, K-fertilizer and 50% N-fertilizer were applied once as the base fertilizer, and the remaining N-fertilizer was applied in the jointing stage	Irrigated by Yellow River water, and the salinity was 0.608 g·L^−1^. Irrigation was done for 3 times during growth period. The irrigation quota for a single was 135 mm. Gasoline pump was used to quantitatively extract water from the canal
BN0	Treatment B: Surfaces were covered with 5 cm thick straw in rows after film-coated planting	0(N0)		
BN1		135(N1)		
BN2		180(N2)		
BN3		225(N3)		
SN0	Treatment S: Straw layers with 5 cm thicknesses were buried at 35 cm below the ground	0(N0)		
SN1		135(N1)		
SN2		180(N2)		
SN3		225(N3)		

### Plant materials

The summer maize used in this study’s experiment was Junkai 918, which was locally grown varieties and sown using a mechanical seeding process at early May and harvested in late September. The spacing of the summer maize plants was 0.35 m apart, and the row spacing was set as 0.45 m.

### Sampling materials

#### Root sample collection

Three representative plants were randomly selected at elongation stage, silking stage, and maturity stage, respectively, and the Monolith 3D spatial sampling method was used to collect the maize root samples^24^. The above-ground plants and root samples were placed at 105 °C and dried at 80 °C to a constant weight, then reweighed. The quality of above-ground dry matter and root samples was measured. The root samples were scanned using an Epson Perfection 4870 root scanner, and the root lengths and other relevant data were analyzed using the Win RHIZO Program.

#### Crop indicators

The yields of the summer maize and its components were measured during the harvesting period. During the harvesting processes, five upper parts of the maize plants were randomly collected from the field, and the stalks, stems, leaves, and seeds were collected. Then, the above-ground plant parts were placed in an oven at 105 °C for 30 min. The oven temperature was then adjusted to 80 °C, and the samples were a constant weight. At that point, the dry matter was reweighed. The samples were then crushed and sifted. The total nitrogen content was determined using a Kjeldahl Method via an H_2_SO_4_/H_2_O_2_ boiling process.

#### Nitrogen use indicators^25^

The *partial factor productivity of the applied nitrogen* (PFPN, kg·kg^−1^) referred to the harvest crop grain yields by the amount of nitrogen applied per unit. The calculation is shown in Eq. (1):

1 $${\text{PFPN}} = {\text{Y/F}}$$

In the calculation, Y represents the crop yield with N fertilizer, kg·hm^−2^; and F represents N fertilizer inputs, kg·hm^−2^.

The *agronomic efficiency of the applied nitrogen* (AEN, kg·kg^−1^) referred to increases in crop grain yields associated with the amounts of nitrogen applied per unit. The calculation is shown in Eq. (2):

2 $${\text{AEN}} = \left( {{\text{Y}} - {\text{Y}}_{{0}} } \right){\text{/F}}$$

In the calculation, Y represents the crop yields with N fertilizer, kg·hm^−2^; Y_0_ represents the crop yields without N applications, kg·hm^−2^; and F represents N fertilizer inputs, kg·hm^−2^.

The *apparent recovery efficiency of the applied nitrogen* (REN, kg·kg^−1^) also referred to the recovery rate of the applied nitrogen, which was reflected in the proportion of nitrogen absorbed by the plants from the fertilizer. The calculation was as per Eq. (3):

3 $${\text{REN}} = \left( {{\text{U}} - {\text{U}}_{{0}} } \right){\text{/F}}$$

In the calculation, U represents the total N uptake of the above-ground plant parts at harvest in the N fertilized crops, kg·hm^−2^; U_0_ represents the total N uptake by the above-ground plant parts at harvest for the crops without N application , kg·hm^−2^; and F indicates the N fertilizer inputs, kg·hm^−2^.

#### Statistical analysis

All data obtained within the individual years were processed using Excel 2010, presented as mean ± standard deviation (SD, n = 3), and analyzed by IBM SPSS Statistics 20. A one-way ANOVA was applied to check the significance of the treatments during the study period (*P* < 0.05), followed by Tukey HSD's multiple range tests. Excel 2010 was used to draw the graph.

## Results

### Root length densities

**Figure 2:**
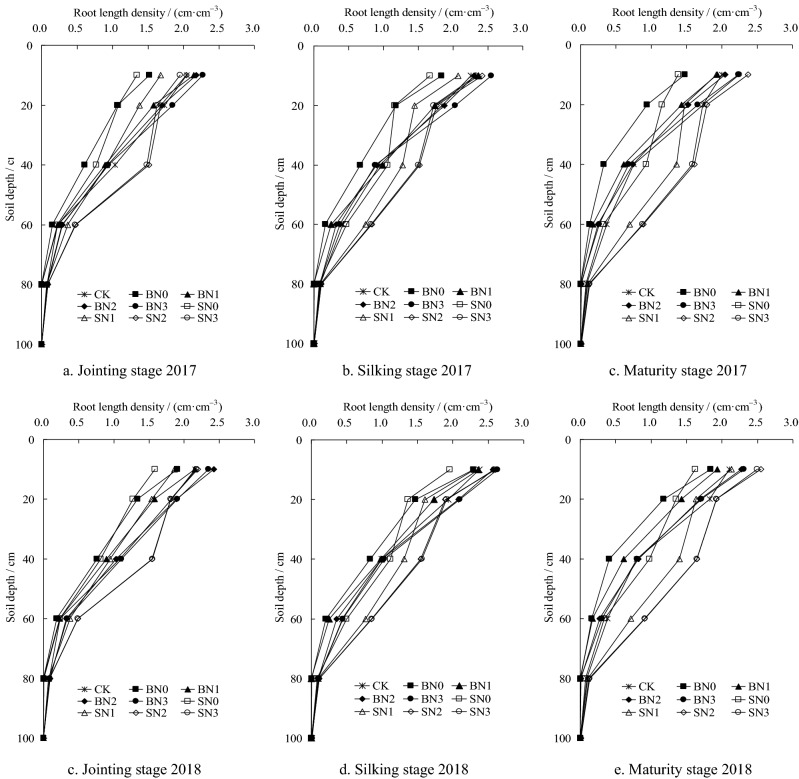
Effects on the RLD of summer maize under different treatments during the growth stages in 2017 and 2018.

The root length density (RLD) was one of important indexes used to study the root systems of crops, and tend to better reflect the distributions of crop roots in soil layers. Since the distribution trend of 2-year RLD was consistent, the statistical index of the following analysis adopts 2-year average value. From the perspective of different straw mulching methods (Fig. 2), the average RLD of Treatment B was determined to have increased by 12.8% and 14.2%, respectively, when compared with Treatment S and CK in 0 to 30 cm soil layers. In 30 to 40 cm soil layers, the average RLD of Treatment S was observed to be significantly increased by 11.7% and 15.8%, respectively, and in the greater than 40 cm soil layers, had increased by 41.7% and 46.5%, respectively, when compared with Treatments B and CK (*P* < 0.05). These results indicated that Treatment B had significantly improved the RLD of 0 to 30 cm surface soil layers. Meanwhile, Treatment S was conducive to deep root growth, particularly in the RLD of greater than 40 cm soil layers. And for the 0 to 30 cm soil layers, it was observed that RLD first increased and then decreased with the increases in N application amounts. The no-nitrogen and low-nitrogen treatments were significantly decreased when compared with Treatment CK, with average decreases of 25.2% and 13.9%. It was also found that the medium N (N2) and high N (N3) treatments only increased 4.9% and 3.8% on average. These findings indicated that low N application amounts significantly reduced the RLD of the crops’ surface root systems, while high N application amounts did not, which was not substantially different from CK treatment results. In the 30 to 40 cm soil layers, the no-nitrogen and low-nitrogen treatments displayed significant decreases when compared with CK treatment, with average decreases of 28.3% and 14.9%, respectively (*P* < 0.05). The medium and high N treatments were found to have increased by 8.7% and 10.3% on average. In the greater than 40 cm soil layers, the RLD of no-nitrogen treatment was found to have decreased by 37.1% on average compared with CK. In the other 3 N treatments, increases of 3.9%, 57.5%, and 56.0% on average were observed. These results indicated that for the deeply buried straw mulching tillage method, the effects of insufficient N applications on the deep root systems were more significant than the effects on the roots closer to the surface. The N2 and N3 treatments both had significant effects on the deep RLD, which was conducive to the growth of deeper root systems.

The RLD of the treatments with no and low-nitrogen applications were found to be significantly decreased when compared with CK at each growth stage. Meanwhile, the RLD of treatments with medium and high N applications increased significantly. In addition, for the greater than 40 cm soil layers, the RLD of each treatment was observed to gradually increase with the growth of summer maize. However, the RLD of Treatments CK and B were found to be significantly decreased at maturity stage. The proportion of RLD in the 1 m soil layer was less than 2.1%. With the exception of SN0, in the greater than 40 cm soil layers, the average RLD of other 3 N application amounts with Treatments S occupied 6.3%, 9.6%, and 10.1% of the 1 m soil layer, respectively. The 3 treatments were found to be significantly increased by 6.8%, 67.5%, and 68.1% when compared with CK. These results indicated that it was beneficial for summer maize root systems to root in a downward direction, and the promotion of the development of deep root systems by the deeply buried straw mulch combined with nitrogen fertilizer tillage method had positive effects on the crops. The RLD of the deep roots had increased with the increases in the nitrogen application amounts. Meanwhile, the differences between the medium and high nitrogen treatments were not found to be significant.

**Figure 3:**
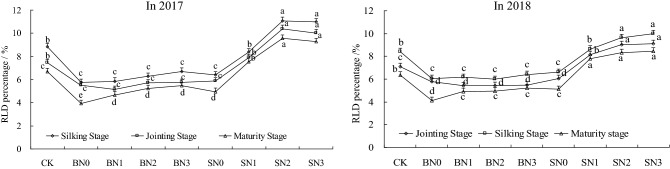
Percentage of RLD in the 30 to 40 cm soil layers of summer maize in 1 m soil layers under different treatments. *Note*: In the Figure, bars with the same letter for the same year indicate that no significant differences were observed at *P* = 0.05.

The RLD and its percentages in the examined summer maize at each growth stage in the soil layers of 30 to 40 cm under different treatments in 2 years are detailed in Fig. 3, respectively. The proportion of RLD in Treatment S was significantly higher than that of Treatments B and CK at each growth stage. With the exception of SN0, the RLD of other Treatments S in the 30 to 40 cm soil layers were determined to have reached between 0.95 and 1.53 cm·cm^−3^. Meanwhile, the RLD of BN2, BN3, and CK treatments during the jointing and silking stages reached 0.8 to 1.08 cm·cm^–3^. However, all of the other treatments had not reached 0.8 cm·cm^−3^. The RLD of the SN1, SN2, and SN3 treatments in the 30 to 40 cm soil layers tended to increase with the growth of the summer maize, while that of other treatments had first increased and then decreased. The RLD in 30 to 40 cm soil layers gradually increased with the increases in the nitrogen application amounts at each growth stage. However, the RLD at the late growth stage of the summer maize tended to be stable under the Treatment B, and the increased effects of the Treatment S was found to be significant, particularly for the SN2 and SN3 treatments. This study analyzed the percentages of RLD in the 30 to 40 cm soil layers and found that the differences of the RLD percentage under the B treatments were not significant, with average decreases ranging between 9.1% and 17.1% when compared with the CK treatment. Therefore, it was indicated that the effects of straw-covered surface tillage method combined with N fertilizer on the improvements of deep root growth had not been significant. With the exception of SN0, the other treatments of Treatment S had increased by between 12.3% and 25.3% on average when compared with CK treatment in 2 years.

### Nitrogen uptake and nitrogen use efficiency

**Figure 4:**
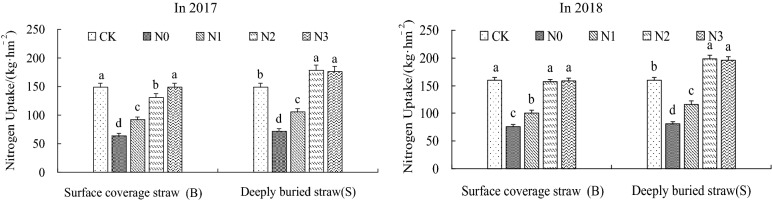
Effects of N application with different straw mulching modes on N uptake at maturity stage of summer maize. *Note*: In the Figure, bars with the same letter for the same year indicate that no significant differences were observed at *P* = 0.05.

**Figure 5:**
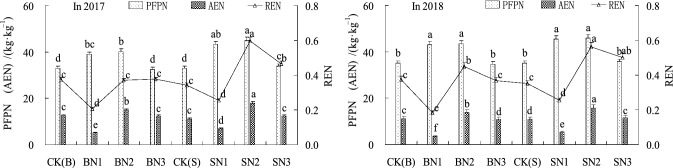
Effects of N application with different straw mulching modes on the nitrogen utilization efficiency at maturity stage. *Note*: In the Figure, CK(B) represents the nitrogen utilization index calculated by the CK under Treatments B and S . The bars with the same letter for the same year indicate that no significant differences were observed at *P* = 0.05.

From the perspective of N application amounts (Fig. 4), the summer maize plants’ N uptake was found to increase with the increases in N application amounts, while the rate of increase was observed to gradually decelerate over time. From the perspective of the different straw mulching methods, the N uptake of the plants in Treatment S significantly increased when compared with that in Treatment B at the same N application dosage, with average increases of 9.2%, 15%, 32%, and 21% in 2 years (*P* < 0.05). During the 2 investigative years, the maximum plant N uptake of Treatments B was observed the BN3 treatment, which had increased by only 0.21%. Meanwhile, the maximum plant N uptake in the S treatments was observed in the SN2 treatment, which increased by 20.4%. Therefore, the plant N uptake could be significantly increased by SN2 treatment in deeply buried straw mulch tillage method with significantly reduced N application amounts. The N uptake of the summer maize plants in 2018 was determined to have increased by between 6.7% and 19.9% when compared with that of 2017. The largest increases were achieved by BN0 and SN0 treatments, which were 19.9% and 14.0%, respectively. This may have been caused by the heavy rainfall which occurred in 2018, and the detailed effects of water on plants N uptake abilities will require further study. When comparing the results detailed in Fig. 4 with those of Fig. 5, it seen that the change trends were consistent between Treatments S at the maturity stage in the 30 to 40 cm soil layers in regard to RLD and N uptake of plants. Therefore, it was clear that Treatments S had significantly increased the root growth of the 30 to 40 cm soil layers, which was a key factor in the plants’ N uptake. The N uptake of the plants were observed to increase with the improved deep root growth. Meanwhile, Treatments B had only improved RLD in the 0 to 30 cm soil layers, which limited the effects of increasing the plants’ N uptake.

With increases in N application amounts, the PFPN, AEN, and REN were observed to first increase and then decrease (Fig. 5) in the overall performance. From the perspective of N application amounts, BN1, BN2, and BN3 treatments of the PFPN had increased by 20.9%, 23.5%, and 0.9%, compared with CK(B). The PFPN of SN1, SN2, and SN3 treatments were observed to have increased by 30.8%, 34.1%, and 2.7%, compared with CK(S). These results indicated that the PFPN differences were not significant when the straw mulching tillage with low and medium N application amounts was conducted, and that they were significantly higher than with CK treatment (*P* < 0.05). However, the differences between CK and straw mulching treatments with high N application amounts were not significant. From the perspective of different straw mulching methods, the PFPN at the same N application amounts in Treatments S displayed average increases of 8.6%, 8.6%, and 3.6%, respectively, when compared with Treatments B. At the same N application amounts of the AEN under the different treatments, the AEN values of Treatments S were higher than those observed for Treatments B, with the rate of increase first increasing and then decreasing with the increases in the N application amounts. The SN2 treatment was found to be the largest, being 52.8% higher than CK treatment. The change trends of the REN and AEN were consistent, and SN2 treatment was also determined to be the largest at 66.8% .

The analysis results detailed in Figs. 3 and 5 showed that the change trends of RLD in the 30 to 40 cm soil layers under the conditions of the deeply buried straw mulch S treatments were consistent with the PFPN, AEN, and REN at the maturity stage. It was found that with the increases in the RLD, the PFPN, AEN, and REN values had first increased and then decreased. However, at the maturity stage, there were no significant differences observed between the medium and high nitrogen applications in the deeply buried straw mulch S treatments in regard to root length densities. However, the PFPN of the SN2 treatment had significantly increased by 30.6%, 41.2%, and 20%, compared with the SN3 treatment. This study’s results clearly showed that the high nitrogen application amounts had ensured plant root growth, yet the nitrogen use efficiency had declined. Therefore, it was confirmed that root growth was promoted with appropriate nitrogen application amounts, which increased the nitrogen use efficiency. Meanwhile, those benefits were not only made invalid with excessive nitrogen application amounts, but pollution risks had resulted in the agricultural ecological environment.

### Summer maize yields

Ear lengths, ear thicknesses, and 100-grain masses are the main agronomic traits of summer maize, which directly affect the yields and Harvest Index (Table 2). The ear lengths and ear thicknesses were not significantly different at the same N application levels in 2 years. The ear lengths and ear thicknesses of Treatments B had increased with the increases in N application amounts. While, the aforementioned of Treatments S had first increased and then decreased. Within the medium and high N levels, the ear lengths and ear thicknesses of Treatments B were not significantly different from those of CK treatment in 2 years. The average ear lengths and ear thicknesses of Treatments S were found to be significantly increased by 11.5% and 8.3%, respectively (*P* < 0.05).

**Table 2:** Effects of the HI and yields of the summer maize under different treatment conditions.

Years	Treatments	Cob Length/cm	Cob Diameter/cm	100-grain mass/g	Yield/(kg·hm^−2^)	Harvest index
2017	CK	20.47 ± 1.02 cd	4.95 ± 0.25a	29.69 ± 1.43ab	7384.96 ± 369.25a	0.37 ± 0.019abc
	BN0	15.66 ± 0.78e	3.91 ± 0.20c	23.43 ± 1.08d	4548.95 ± 227.45c	0.32 ± 0.016c
	BN1	17.67 ± 0.88de	4.26 ± 0.21bc	25.70 ± 1.26bcd	5245.97 ± 262.30bc	0.33 ± 0.017bc
	BN2	20.13 ± 1.00 cd	4.92 ± 0.25ab	28.59 ± 1.32bc	7248.33 ± 362.42a	0.36 ± 0.018abc
	BN3	20.61 ± 1.03bc	5.05 ± 0.25a	28.60 ± 1.43ab	7315.27 ± 365.76a	0.38 ± 0.019ab
	SN0	16.01 ± 0.80e	4.12 ± 0.20c	24.78 ± 1.17 cd	4879.98 ± 244.00c	0.33 ± 0.017bc
	SN1	19.86 ± 0.99 cd	5.02 ± 0.25a	27.35 ± 1.30bc	5836.52 ± 291.83b	0.35 ± 0.018abc
	SN2	23.62 ± 1.18a	5.43 ± 0.27a	31.38 ± 1.56a	8103.08 ± 405.15a	0.40 ± 0.020a
	SN3	23.42 ± 1.17ab	5.38 ± 0.27a	30.37 ± 1.57a	7633.30 ± 381.66a	0.38 ± 0.019ab
2018	CK	22.40 ± 1.12ab	5.17 ± 0.29bc	30.12 ± 1.51ab	7599.60 ± 379.98a	0.38 ± 0.019abc
	BN0	17.50 ± 0.88c	4.78 ± 0.24d	21.53 ± 1.08d	5351.70 ± 267.59b	0.32 ± 0.016d
	BN1	18.80 ± 0.94c	5.08 ± 0.25c	26.52 ± 1.33bc	5828.85 ± 291.44b	0.36 ± 0.018bcd
	BN2	21.43 ± 1.13ab	5.23 ± 0.26bc	28.86 ± 1.44ab	7525.35 ± 376.27a	0.39 ± 0.019b
	BN3	22.90 ± 1.15a	5.37 ± 0.27b	30.11 ± 1.51ab	7730.55 ± 386.53a	0.40 ± 0.020abc
	SN0	17.23 ± 0.94c	4.93 ± 0.25 cd	24.65 ± 1.23 cd	5422.20 ± 271.11b	0.34 ± 0.017 cd
	SN1	19.63 ± 0.98bc	5.12 ± 0.26c	28.19 ± 1.41bc	6143.70 ± 307.19b	0.38 ± 0.019abc
	SN2	24.10 ± 1.21a	5.54 ± 0.28a	32.78 ± 1.64a	8268.45 ± 413.42a	0.43 ± 0.022a
	SN3	23.90 ± 1.20a	5.49 ± 0.27a	32.89 ± 1.64a	8035.05 ± 401.75a	0.42 ± 0.020ab
**F value of ANOVA analysis**						
Straw Mulching		17.2**	5.4**	20.4**	8.0**	5.4**
Nitrogen Applications		52.3**	16.0**	60.4**	147.4**	22.3**
Straw Mulching × Nitrogen Applications		2.8*	3.3*	10.9**	11.3**	4.2*

The * represents significant (*P* < 0.05), and ** represents extremely significant (*P* < 0.01).

The values in the same column and same year followed by different letters indicate significant differences were observed, *P* < 0.05.

The 100-grain mass values of Treatments B with high N application amounts (BN3) displayed no differences with CK treatment, and the other treatments had displayed significantly reduced. While,compared with CK treatment, the 100-grain mass values of the medium and high N application with Treatments S were found to be significantly increased by 8.8% and 9.3% (*P* < 0.05), and the other treatments were significantly reduced. Under different N application levels, the summer maize yields of Treatments S had average increases of 4.3%, 8.8%, 10.8%, and 4.1%, respectively, compared with Treatments B. At the same N application level, the yields undergoing Treatments S were significantly higher than that of Treatments B, and had increased significantly with the increases in the N application amounts. The summer maize yields of Treatments B had increased with the increases in the N applications amount, then the increase range gradually decreased. The yield of high N (BN3) treatment were found to be the highest, and the effects of the increasing yields were not significant. The summer maize yields in Treatments S first increased and then decreased, and the medium N treatment (SN2) was the largest. The yields of SN2 significantly increased by 9.3% on average. The analysis results detailed in Fig. 3 and Table 2 reveal that the variation trends of RLD in the 30 to 40 cm soil layers under the conditions of Treatments S were consistent with yield. Therefore, Treatments S had increased the absorption abilities of water and nutrients in the root systems by increasing the deep RLD, thereby increasing the yields. While Treatments B had significantly increased the RLD in the 0 to 30 cm soil layers, and had limited effects on the extraction of water and nutrients in the deeper soil. Furthermore, the aforementioned treatments had little effect on the growth rates and yields during the medium and later crop growth stages.

The Harvest Index (HI)^26^, which can be significantly affected by crop panicle morphology and N application levels, represents the ratios of the grain mass of crop panicle to the dry matter mass of above-ground parts, and reflects the proportions of the assimilation products in grains and vegetative organs. Under the straw mulching treatments with different N application amounts, the HI varied between 0.32 and 0.42, with significant differences observed among the treatments (*P* < 0.05, Table 2). These results indicated that HI was susceptible to N applications and straw mulching methods. Under different N application conditions, the average values of HI in Treatments S had increased by 5.0–10.3% when compared with Treatments B. With increases in N application amounts, the HI of Treatments B increased, then the range gradually decreased over time. And the HI under the high N application treatments (BN3) reached a maximum, which was 4.5% higher than that of Treatment CK on average. With increases in N application amounts, the HI of Treatments S had first increased and then decreased. The HI with the medium N applications (SN2) reached a maximum, which was 10.7% higher than that of Treatment CK on average. Therefore, the results obtained indicated that the HI could be significantly improved using the deeply buried straw mulching method, and the effects obtained using the medium N level treatment (SN2) was more significant.

## Discussions

It was determined that the crop root systems were the key to improving the nutrient absorption rates, which was a key factor for high crop yields^27^, particularly the deep root (> 30 cm), which tend to be affected by the growth environment, fertilization, and other factors^5,10^. The RLD was one of the important indexes for studying crop root systems. The results showed that Treatments B had significantly (*P* < 0.05) increased RLD of the surface soil layers (0 to 30 cm), with average increases of 14.2% in 2 years compared with CK treatment. While, Treatments S had significantly increased RLD of deep soil layers, with average increases of 46.5% observed. Under different straw mulching treatments, the RLD had first increased and then decreased with the increases in N application amounts. Barraclough^28^ found that when RLD was less than 0.8 to 1.0 cm·cm^−3^, the absorption abilities of water and nutrients by crop roots would be influenced. Under Treatments S conditions, with the exception of SN0 treatment, the effects tended to increase during the later growth stage, and the percentage of RLD (> 0.8 to 1.0 cm·cm^−3^) increased significantly. These indicated that deeply buried straw mulch combined with N application in arid areas was more beneficial to deep roots. Generally speaking, the overall effects of SN2 treatment was the most significant.

The results showed that the N uptake abilities of summer maize plants under Treatments S were significantly higher than those under Treatments B. Also, the plant N uptakes were found to be increased within an appropriate range of N application amounts. The N uptake of SN2 treatment was the largest, with an average increase of 20.4% observed when compared with CK treatment. There were consistencies observed between the change trends of the deep RLD and the plants’ N uptake, with the deep RLD found to be increased by the improved N uptake abilities. Treatments B had significantly increased the RLD of the surface soil layers, while the effects of the increased plant N uptake were limited. These findings were similar to the conclusions previously reached by Kang et al.^8^. The effects of the same N levels in Treatments S were found to be better than those of Treatments B. Both types of straw mulching had good effects when combined with medium N application (N2), with SN2 treatment found to be the most effective. The PFPN, AEN, and REN of SN2 had increased by 34.1%, 52.8%, and 66.8% on average (*P* < 0.05) compared with CK treatment. The change trend of SN2 during the maturity stage was consistent with the change trend of the deep soil RLD. Although high N application amount could guarantee the nutrients needed for the growth of plant roots, they could not fully absorb, which reduced the N utilization rates, and resulted in pollution of the agricultural ecological environment. Therefore, it was ascertained that developed deep root systems was one of the key factors for improvements in the N utilization efficiency of crops in arid areas.

N fertilizer applications are one of the important measures used to increase crop yields. The formations of crop yields require certain amounts of nitrogen fertilizer, and insufficient supplies of N fertilizer will lead to crop losses^29,30^. Meanwhile, excessive supplies will lead to decreases in N utilization rates, which will in turn cause significant reductions in crop yields, and pollution risks in the ecological environments^31^. Under Treatments B, the crop yields were significantly increased with increases in the N application amounts, while the yield increases were found to gradually decrease over time^32^. The summer maize yields of treatments with high N application (BN3) had only increased 0.4% on average when compared with CK treatment. This was due to the fact that the “low temperature effects”^4,22^ caused by the Treatments B had affected the development of summer maize during the seedling stage. The “low temperature effects” delayed the growth of the underground crop parts, and also inhibited the N absorption and dry matter transference after the flowering stage^5^. However, due to the special “cooling effects” of the straw-covered surfaces, it was found to be beneficial to improve the root activities during the middle and later growth stages, in order to delay the plants aging processes. Therefore, the crop yields could be stabilized under high N application (BN3), but the increasing yield effects were not significant. Generally speaking, the results of Treatments B showed that the effects on summer maize yields were significantly positively correlated with the N application amounts (*P* < 0.05; *R*^2^ = 0.81), which was consistent with the conclusions of Jiao et al.^33^. However, the conclusions were significantly different from the results of Treatments S in this study. The summer maize yields and yield components examined under Treatments S were observed to first increase and then decrease with the increases in N application amounts. The SN2 treatment showed the largest effects, with an average yield increase of 9.3% compared with CK treatment. The yields were found to be consistent with the variation trends of RLD in the deep soil layers, which were significantly positively correlated (*P* < 0.05, *R*^2^ = 0.97). However, there were no significant relationships observed between the yields and the total RLD. It could be seen that developed deep root systems were the key to high yields in arid crops.

It was recommended that the following goals be achieved for summer maize in Hetao Irrigated District: 1. The redundant roots of the surface soil must be properly eliminated to reduce the consumption of soil moisture during the early growth stages to the greatest extent; 2. The role of plant root systems in carrying water will be enhanced^34^ by improving the deep soil RLD. The spatial dislocations of nutrients and water could be relieved in a certain extent, and the coupling of water and nutrients in space could be effectively realized; 3. The promotion of root development should be achieved with appropriate fertilizer applications to regulate the water and fertilizer in the root systems, which would increase summer maize root systems absorbing nitrogen and water in the deep soil layers. In this way, both N utilization efficiency and yields would be improved. This study suggests that it will be beneficial to achieve the aforementioned goals by the deeply buried straw mulch combined with medium N application (SN2).

## Conclusions

In this study, the dynamic responses of summer maize yields, root length distributions, and N utilization rates to the interactions between straw mulching and N application were preliminarily revealed. The most effective treatment was the deeply buried straw mulch combined with medium N fertilizer application (SN2), which increased deep root length densities by 67.5%, enhanced N use efficiency by 66.8%, and increased in production by 9.3% when compared with CK treatment. In addition, the summer maize root system distributions were improved. Therefore, the results obtained in this study provided certain theoretical information for the effective utilization of straw and rational N application practices in the future in Hetao Irrigation District.

However, under straw mulching model, there were still many aspects worthy of attention. For example, the effects on grain yields of the depths and locations of N applications when combined with straw mulching and irrigation processes require investigation to improve summer maize quality and ecological environments. In addition, the coupling effects of water-fertilizer should be analyzed and coupling models of water-fertilizer established, which should include the important soil nutrient factors following straw mulching processes.
